# Augmented Intelligence in Joint Replacement Surgery: How can artificial intelligence (AI) bridge the gap between the man and the machine?

**DOI:** 10.1186/s42836-021-00108-1

**Published:** 2022-02-02

**Authors:** Vaibhav Bagaria, Anjali Tiwari

**Affiliations:** Department of Orthopaedic Surgery, Sir H.N. Reliance Foundation Hospital and Research Center, Girgaon, Mumbai, Maharashtra India

**Keywords:** Artificial intelligence, Arthroplasty, Robotics, Machine learning, Interface

## Abstract

Robot-assisted arthroplasty is likely to grow exponentially in the years to come. While most surgeons recognize their superiority in refining alignment and improving component positioning accuracy, the universal adaptability of robots remains slow due to certain technological and behavioural gaps. Endoprosthesis robots currently suffer from increased reaction time, lack of natural adaptation to the surgeon's abilities, and unavailability and inapplicability in different surgical contexts. As humans and machines have to move forward into the next phase of their relationship, robots enabled with artificial intelligence (AI) may become more advanced and an alternative to overcome existing challenges like cost, training, and improve performance based on feedback provided by surgeons. Augmented intelligence is perhaps a more apt word than artificial, as it reflects more human-machine fusion and several areas are already proactively adopting the terminology. Arthroplasty robots can benefit from AI by using computer vision models, applying sensors, and integrating feedback and loop execution. All of this would help achieve a technical superiority to the surgeon alone. This brief perspective on how humans and machines are likely to benefit from the integration of AI outlines the technical side of this enablement.

## Background

### Augmented Intelligence

There is no field in medicine that stays outside of AI. Orthopedics among all medical specialities is a special kid as all orthopods (an orthopedic surgeon) love their toys and now they have a new tool: robots. It is estimated that robotic arthroplasties will outnumber manual jig-based arthroplasties in a decade. Even though robotic arthroplasty has not yet proven its superiority in terms of long-term clinical results, the technology adoption has been rapid and universal [1, 2]. Important concerns linger and are mainly related to operation and response time, ease of use, cost, and level of autonomy. This is where augmented intelligence probably comes in, providing the data points to ensure that machine learning algorithms enable next-generation robots to learn, unlearn, and execute procedures efficiently and safely. AI tools to support medical decisions are now an established norm. The use of artificial intelligence to improve robotic technology is advancing rapidly by enabling a more autonomous robotic system capable of performing knee and hip arthroplasties in a manner that requires minimal human intervention.

Alan Turing's theory of computation suggested that all forms of computation can be described digitally, and put together a series of computations that lead to the formation of algorithms. Surgeons are constantly refining their decisions by improvising the algorithm based on their results and experience. This algorithmic improvisation is based on recognition, reasoning and communication. In terms of surgery, it consists of preoperative planning, intraoperative execution, assessment of complications, and monitoring outcomes from short- and long-term results.

Surgeons learn from their mistakes and adapt. AI will add this very special dimension to robotics. Enabling robots to adapt and learn from their mistakes will be the primary goal of augmented intelligence. The success and feasibility of the program depend on the simulation of the exact conditions that surgeons experience in everyday life. These experiences and feedbacks from experts then reinforce AI-enabled robots with continuously accumulated knowledge to intuitively make better decisions.

So how exactly would robots be expanded? Robots can benefit from AI by using the computer vision model, applying sensors, and integrating feedback and loop execution, all of which would help achieve technical superiority to the surgeon alone.

## Computer Vision Model

Datasets can be used to help robots recognize objects and take actions to deliver the desired results [3]. Currently, the robotic platform takes limited sets of data from probe sensors or CT (Computed Tomography) data. Robot extrapolates them to create a model that they cannot see, just evoke a software-based image. Artificial Intelligence tools set helps them to process and model solutions to better identify and understand human anatomy. This ensures that a tailor-made solution is developed for each human phenotype based on a large data set with an extensive data training exercise.

## Application of Sensors

Sensors aid robots to perceive the visuals of the environment in a way much similar to the five key senses of human beings [4]. This is facilitated by using combinations of various sensing technologies like:

- Optical sensors
- Temperature and humidity sensors
- Ultrasonic sensors
- Vibration sensor
- Millimetre-wave sensors

Surgical robotic likely involves a variety of sensors to collect real-time data related to the surgery [5]. These robots will improve the quality of the data and, with the help of increased augmented intelligence, will help with feedback and improvisation of performance as defined below.

We are now on the stepping stone of the emergence of third-generation computers, to which scientists are referring as Augmented Intelligence. Augmented Intelligence is a subset of Artificial Intelligence technology that mimics human cognitive abilities like memory and sequencing, perception, anticipation, problem solving, and decision making in software. Augmented Intelligence systems find hidden and untraceable meaning within the data which is beyond the capability of normal humans. Thus transform user interaction by providing the right advice, with correct evidence and results at the right time for any context [6].

In this coming era, systems will be big data-based, identifying and extracting the meaning of the faded information, constantly learning and producing outputs that expand human cognitive function by pairing humans and machines. Augmented intelligence may potentially reinvent every business and medical process in the coming years with user experience and directly impact the business model. These future systems based on augmented intelligence may emulate human cognitive processes in a computerized model and learn with each user and each data interaction, as opposed to current rule-based decisioning using current command-driven computers.

## Major Developments across the globe

Talking about the related work in the industry, medical robotics has so far proven to be the most successful in surgical and rehabilitation (including exoskeletons) to date. The market-leading robot for soft tissue tele-manipulators is the da Vinci (Intuitive Surgical) robot. Currently, 5,000 robots are used in around 1 million operations per year, mainly in the fields of urology and gynecology. Some of the established AI-enabled robots are TransEnterix-SurgiBot, Senhence, a robot-assisted surgical system, Coridus Vascular Robotics (cardiology robot CorPath GRX), Artas, a major robot platform for hair implantation, endoscopic Monarch from Auris Health, orthopedic Renaissance by Mazor Robotics, Medtronic-HUGO (Einstein) and Medrobotics-FLEX robotic flexible endoscopic surgical procedures. In 2018, the Corindus-CorPath GRX platform received approval from the US Food and Drug Administration (FDA) to develop the first automated robot movement called "Rotate on Retract (RoR)”. This is regarded as a primitive step towards autonomous robotic operations [7, 8]. In orthopedics, the commonly used robots include Navio, Cuvis, Rosa, CORI and MAKO. However, all the robots in absence of incorporated AI suffer from distinct disadvantages. As with any technology upgrade, robotics and AI have their demerits. Firstly, the cost of surgery escalation. Surgical robots are costly to maintain, and their operation requires additional training, which is also expensive. Secondly—it is the issue of latency *i*.*e*. the time robot takes to carry out the surgeon's commands. It has been observed in current robotic bots that it takes a few moments for the computer to communicate with the robotic arms. While this is not a major issue for regular surgeries, but a serious concern for surgeons to respond quickly to problems that occur during the surgeries. One of the key approaches proposed in this paper is to improve the latency by incorporating feedback and execution loop. Thus the issue of latency can be addressed instantly while with more commercial use and awareness improvement of such devices will reduce the cost of manufacturing, as well as maintenance in coming future. Upcoming technological integrations, such as the use of 5G Telecom too, maybe a step in this important direction [9].

## Feedback and Execution Loop

The feedback and execution loop technique is considered an established management strategy to ensure that all team members are in the same loop. With AI, this feedback loop ensures the identification of areas of improvement and learning. These potential activities are then translated into actionable work by Robot and result in a more coordinated, engaged, and collaborative outcome. In AI, this has been made possible by the use of the technique: deep convolutional neural networks, inspired by nothing less than a visual stream of primates residing in the neocortex of the human brain.

**Fig. 1:**
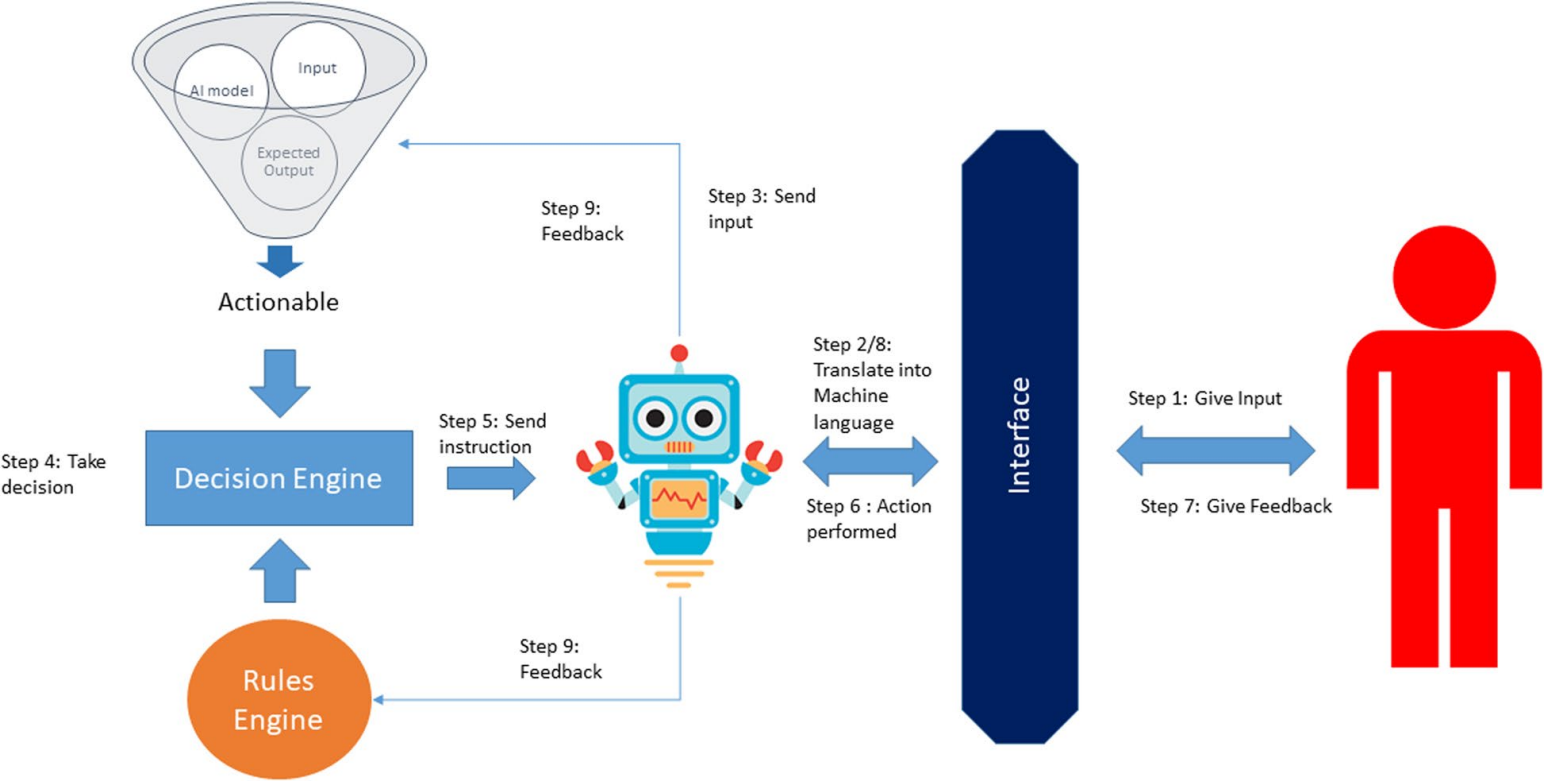
Collaboration of Robotics and Artificial Intelligence

The high degree of spatial and temporal accuracy, which is the hallmark of the human brain, needs to be imitated by the surgical robots to become increasingly independent. In the initial stages, surgeons should hold them in their hands like a surgical trainee to ensure mistakes are recorded. Instead, credible steps are taken to ensure mistakes are not repeated in the future (Fig. 1).

The surgeon interacts with the machine interface to provide inputs. This entry is extracted by robot in a machine-readable format. This robot shares it with the actionable AI model. With the model, the actions which could be learnt based on continuous interactions can be fed into the machine and thus machine takes actions accordingly. Thus robot learns about the output of the model and the rules or guidelines that govern its characteristics. Humans can give feedback for an action that is carried out by the robot. This feedback is sent to both the AI model to change actionable results. The real difference between the traditional robots and robots with the built-in artificial intelligence engine is the ability to understand the feedback and improve the result next time, rather than give the same result for input as the simple robotic system does.

## Conclusion

*Mind the gap*! The missing piece to enable heralding an era of true Autonomous Surgeon Robots (Surgi-bots) seem to be the incorporation of augmented intelligence into surgical robots.

Many companies have put AI as a special feature in their system but currently, this is far from credible and is mainly a commercial selling gimmick. Virtual reality (VR) is a new technology that can teach surgeons new procedures and determine their skill level before performing surgery on patients. In addition, virtual reality enables the surgeon to simulate surgeries and to return to the same procedure or task several times later to optimize their actions. Thus AI bot and VR technology will go hand in hand to improve the surgical experience. To get into the operating theatres more autonomously, the surgical robots of the future must be held in the hand of experienced surgeons and surgical trainees and, like the human brain, augmented intelligence and virtual reality must be enhanced to ensure this bridges the gap between man and machine!

## Abbreviations

AI: Artificial intelligence
CT: Computed tomography
